# Genomics: The Year of the Rat

**DOI:** 10.1289/ehp.112-1247663

**Published:** 2004-11

**Authors:** Ernie Hood

After two years of intensive efforts by an international consortium of researchers, the Brown Norway rat (*Rattus norvegicus*) joins the human and the mouse as the third mammalian genomic sequence to be completed. The achievement is expected to yield important new knowledge about mammalian evolution and human disease processes, and should also contribute significantly to progress in toxicogenomics.

The project, funded primarily by the National Human Genome Research Institute (NHGRI) and the National Heart, Lung, and Blood Institute (NHLBI), was conducted by the Rat Genome Sequencing Project Consortium. The Human Genome Sequencing Center at Baylor College of Medicine led the collaboration, assembled the genome, and coordinated the data and resource contributions of a large network of academic and private research centers. Next, an international team comprising more than 20 groups in six countries analyzed the results vis-à-vis the human and mouse genomes. The sequence was published in the 1 April 2004 issue of *Nature*, along with more than 30 papers analyzing the results relative to the human and mouse genomes published simultaneously in the April 2004 issue of *Genome Research.*

The Brown Norway rat has long been one of the primary models employed in biomedical, toxicological, and pharmaceutical research. “A large number of human diseases are mimicked in the rat model, and having the genome sequence lets us easily walk between what we understand in the physiology and biology in the rat, and translate that to a better understanding of human biology and disease processes,” says Susan Old, associate director of the Clinical and Molecular Disease Program in the NHLBI Division of Heart and Vascular Diseases and a project officer for the sequencing initiative. She adds, “Our hope is to improve the health of the individual by better understanding of the mechanism of disease, and to develop better therapeutics and diagnostics. We are going to be able to do that a lot more effectively than we had been able to previously.”

The availability of the rat genome sequence should also have a profound impact on toxicogenomics. “At the present time, when we generate expression profiles that are associated with a particular disease or genotype, or in response to a toxicant, we still have difficulty putting together the story of what genes and what pathways are being affected, because a lot of the [genes] represented by features on the DNA micro-arrays have still not been identified,” says Helmut Zarbl, a toxicologist at the Fred Hutchinson Cancer Research Center in Seattle. Knowing the locations and identities of all of the genes in the rat genome will aid toxicogenomicists in their efforts to accurately characterize their functions—“to actually put the story together and come up with the predictive toxicology we’re looking for,” says Zarbl.

Zarbl’s group uses rat models to search for genes associated with human breast cancer. He says having the sequence in hand will advance his work as well as toxicogenomics studies of many other complex diseases strongly suspected to be linked to gene–environment interactions. “By being able to map some of these complex diseases in the rat model and find the causal genes,” he says, “we can then very quickly go to human studies using comparative sequence analysis to formulate hypotheses about what genes are involved in human disease.”

According to Michael Waters, assistant director of database development at the NIEHS-based National Center for Toxicogenomics (NCT), having the sequence will enhance work being conducted in a variety of “omics” areas. “We at the NCT are using microarray technology, but we’re also using proteomics, and we want to be able to use metabolomics to understand how toxicants operate, what their mode of action is, what some of the biomarkers are that would indicate when a toxic outcome is likely to occur, [and] . . . predict those effects at earlier times and lower doses,” he says. Waters says the rat genome information provides the genetic scaffold for scientists to link expression profiles to a genome that is relevant to toxicology.

With the rat, mouse, and human genome sequences now completed, comparative genomics—identifying the essential functional and structural components of the human genome by comparing it with the genomes of other organisms—is positioned for rapid acceleration. “Every model organism has its advantages and disadvantages, and the more of them that we have to do experiments in, the more quickly we’ll be able to find genes and genotypes associated with specific phenotypes, relate these genotypes back to the human genome, and find causes of human diseases,” says Zarbl.

In August 2004, NHGRI announced that it has added 18 new model organisms to its sequencing pipeline, including the orangutan, the African savannah elephant, the rabbit, and the domestic cat. Other groups are working on sequencing the dog, the cow, the macaque, and several nonmammalian species. Many of these projects are expected to be completed within a few years.

Waters anticipates that the flood of additional genomes will be extremely valuable to toxicogenomics in two specific ways. First, he says, the value of existing databases will be enhanced, with the cross-species genomic information contributing to chemical risk assessment in the ecological domain, as well as in human health. Second, we could possibly learn far more about basic biological function in terms of phylogenetic relationships.

## Figures and Tables

**Figure:**
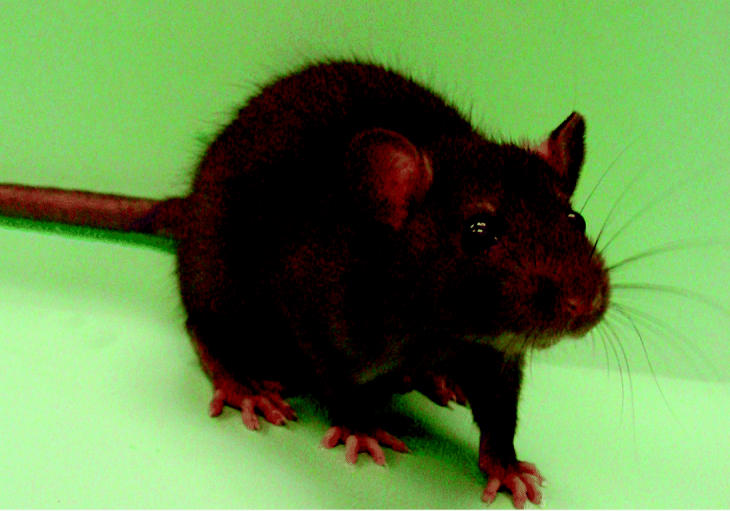
**Three’s company.** The Brown Norway rat joins the human and the mouse as the third mammal to have its genome fully sequenced.

